# Electrodynamics Sensor for the Image Reconstruction Process in an Electrical Charge Tomography System

**DOI:** 10.3390/s91210291

**Published:** 2009-12-18

**Authors:** Mohd Fua'ad Rahmat, Mohd Daud Isa, Ruzairi Abdul Rahim, Tengku Ahmad Raja Hussin

**Affiliations:** 1 Control and Instrumentation Engineering Department, Faculty of Electrical Engineering, Universiti Technologi Malaysia, 81310 Skudai, Johor Bharu, Johor, Malaysia; E-Mail: ruzairi@fke.utm.my; 2 Department of Electrical Engineering, Politeknik Kota Bharu, KM.24 Kok Lanas, 16400 Ketereh, Kota Bharu, Kelantan, Malaysia; E-Mail: peppkb@yahoo.com; 3 Department of General Studies, Politeknik Kota Bharu, KM.24 Kok Lanas, 16400 Ketereh, Kota Bharu, Kelantan, Malaysia; E-Mail: tabs2104@yahoo.com

**Keywords:** image reconstruction, electrodynamics sensor, linear back projectios, filter back projectios, least squares with regularization, CCD-Camera

## Abstract

Electrical charge tomography (EChT) is a non-invasive imaging technique that is aimed to reconstruct the image of materials being conveyed based on data measured by an electrodynamics sensor installed around the pipe. Image reconstruction in electrical charge tomography is vital and has not been widely studied before. Three methods have been introduced before, namely the linear back projection method, the filtered back projection method and the least square method. These methods normally face ill-posed problems and their solutions are unstable and inaccurate. In order to ensure the stability and accuracy, a special solution should be applied to obtain a meaningful image reconstruction result. In this paper, a new image reconstruction method – Least squares with regularization (LSR) will be introduced to reconstruct the image of material in a gravity mode conveyor pipeline for electrical charge tomography. Numerical analysis results based on simulation data indicated that this algorithm efficiently overcomes the numerical instability. The results show that the accuracy of the reconstruction images obtained using the proposed algorithm was enhanced and similar to the image captured by a CCD Camera. As a result, an efficient method for electrical charge tomography image reconstruction has been introduced.

## 1. Introduction

Process tomography allows boundaries between heterogeneous compounds and homogeneous objects in a process to be imaged using a non-intrusive sensor. The basic idea of process tomography is to install a number of sensors around the pipe or vessel to be imaged. The sensor output signal depends on the position of the component boundaries within their sensing zones. The output signals are conditioned and sent as input to a computer, which is used to reconstruct a tomography image of the cross section being observed by the sensor. These tomography images have the potential of providing information on concentration distributions in the pipeline, information on flow regimes, velocity profiles, component volume flow rates and particle size measurements. Process tomography has a very good application foreground in industries [1].

An image reconstruction in Electrical Charge Tomography (EChT) is typically an ill-posed problem. The small changes in the data cause arbitrarily large change in the solution, and this is reflected in ill conditioning of matrix sensitivity of the discrete model. The Tikhonov regularization method is an effective method to solve ill-posed inverse problems [2]. The Thikonov method has been applied to electrical capacitance tomography for image reconstruction by Peng *et al.* and Lionheart [3–5]. This regularization of the problem is required to filter out the influence of the noise. A common feature of this regularization method is that it depends on some regularization parameters that control how much filtering is introduced by regularization without losing too much information in the computed solution. The purpose of regularization optimization is to provide an efficient and numerically stable method that will provide a good approximation to the desired unknown solution.

The theory of ill-posed problems is well developed in many papers [6–8]. The Singular Value Decomposition (SVD) method can easily reveal this problem with a Picard chart and the condition number of matrix sensitivity [9]. Regardless of the image reconstruction process, validation of the images is very important. In this system, a digital imaging technique is used to interrogate the flow in a pipeline around the sensing area. When particles are flowing through a pipeline, it is possible to acquire images using a CCD camera with a suitable illumination light source [10]. It is a simple matter to focus a CCD camera on this system and to acquire digital images.

This paper will highlight a discussion on electrodynamics sensor modeling and the functional of the conditional circuit used in this system. On top of that, a new image reconstruction method called Least Squares with Regularization (LSR) will be explained. Previous methods like Linear Back Projection (LBP) and Filter Back Projection (FBP) will also be summarized. The results show the comparison in simulation images and condition number for stability between the new and previous methods introduced for image reconstruction. In the last section, the validation of the image reconstructed by the LBP, FBP and LSR methods with images captured by a CCD camera will be presented. The method that produces images which are similar to the images captured by CCD camera will be identified as the best method for image reconstruction in EChT.

## 2. Electrodynamics Sensor

This electrical charge tomography system uses an electrodynamics sensor to detect the nature of electrostatic charge on the moving particles. This is because an electrodynamics sensor is capable of achieving a higher sensitivity as required in the mass flow rate measurement of dilute-phase solid flow and is less affected by stationary solids accreted on the pipe wall. In order to understand the sensor, a suitable mathematical model is inevitable. The corresponding models have been discussed in detail [11-13]. Basically, the induction model for single-charged particle q can be derived from equation (1):

(1) $$\text{E}=\frac{q}{4\pi r^{2}\varepsilon_{o}}$$

where *E* is the electric field, *ε*_o_ is the permittivity of free space, *q* is the charge of the particle and *r* is the distance between the charged particle and a particular point.

**Figure 1.**
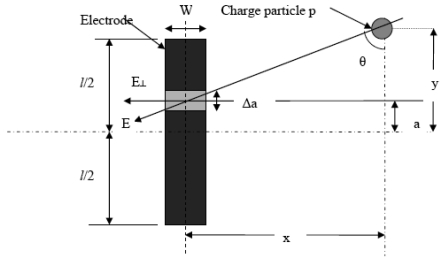
Mathematical model of electrostatic signal on moving particle.

Assume that a particle *p*, carrying a charge *q*, traveling in uniform velocity *v*, along a path which is perpendicular to vertical axis of the electrode. This is illustrated in Figure 1.

From equation (1) and is derivation it shows that the total charge induced into the sensor, *Q* is given by equation (2) [14]:

(2) $$\text{Q}=-\frac{\text{qxW}}{4\pi }\int_{l/2}^{l/2}\frac{dV}{{\left[{\left(\text{y}-\text{a}\right)}^{2}+{\text{x}}^{2}\right]}^{3/2}}$$

where *v* = velocity of particles, *l* = length of electrode, *W* = width of electrode, *x* = distance from electrode to particle in x-axis, *y* = points of particle travel along y-axis, *t* = time taken at any point with velocity *v, a* = length from the center of electrode to the point taken. With *y* = *vt*, then the current, *I* is given by equation (3):

(3) I=dQ/dt

**Figure 2.**
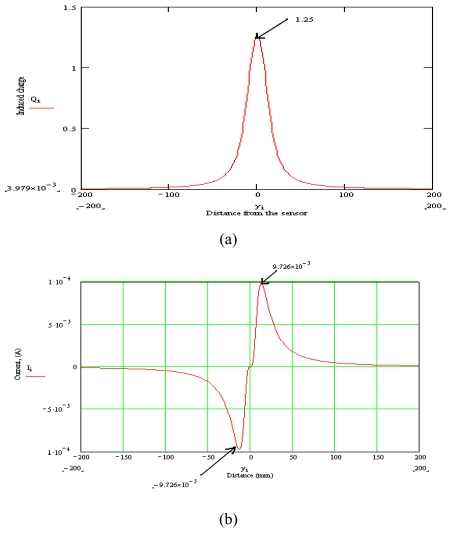
Waveform for (a) Induced charge and (b) Current signal.

Based on the equations (2) and (3), the corresponding waveform of an induced charge on the sensor (*Q*) and the sensor output (*I*) has been plotted utilizing the MathCAD software. The graphical results as shown in Figure 2 are obtained by substituting the values of *q* = 1 Coulomb, *x* = 10 mm, *W* = 10 mm, *l* = 10 mm and *v* = 5000 mms^-1^ into the equations.

**Figure 3.**
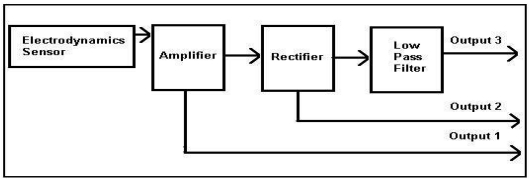
A block diagram of an electrodynamics sensor conditioning circuits.

The electrodynamics sensor plays an important role in the electrical charge tomography system. Figure 3 shows the block diagram of the electrodynamics sensor conditioning circuits. They consist of several parts such as an electrode/electrodynamics sensor, an amplifier, a rectifier and a low-pass filter. The purpose of the electrodynamics sensor is to capture the electrical charge from the conveyed material such as plastic beads that pass through the transducer. The electrical charge detected by the sensor will be converted into a voltage and sent to the image reconstruction system (computer) through the data acquisition card.

Output 1 is an AC signal used for velocity measurement, while output 2 is used for spatial filtering test. Output 3 is a DC averaged voltage and is used for concentration measurement and flow regimes identification. Output 3 is the signal of interest for the proposed system.

**Figure 4.**
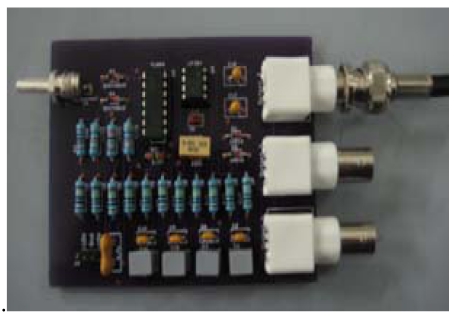
Electrodynamics sensor fabricated in printed circuit board.

Figure 4 shows an electrodynamics sensor fabricated on a printed circuit board. The electrode is a silver steel conductor rod located at the left in Figure 4. The other steel connector rods on the right in Figure 4 are outputs 1, 2 and 3, respectively. The sensor electrode is used to detect the charge on the moving particle, which passes through the pipe.

## 3. Electrical Charge Tomography System (EChT)

### 3.1. Electrical Charge Tomography Measurement System

**Figure 5.**
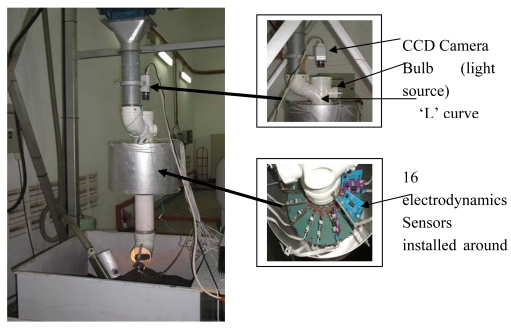
Experiment apparatus for data capturing process.

Figure 5 shows the experimental apparatus for the data and video capturing process by an electrodynamics sensor and CCD camera. The CCD camera is placed above of the test flow rig so that it can capture the image of the solid particle distribution the pipe. The video capturing process will be conducted through a hole in the pipe where the material is being dropped with assistance from the light of a bulb installed at the corner and at the end of the pipe. The electrodynamics sensor is located 10 mm below the ‘L’ curve of the pipe. The material used in this system is plastic beads with the nominal size of 3 mm. The electrostatic charge carried by these particles is induced to the electrodynamics sensor whenever they passed through it. This charge will be converted into a voltage and sent to computer storage via a Keithely STA-1800HC data acquisition card.

The CCD camera will be used to capture the video of material flow in the pipeline. Data from electrodynamics sensors are captured using the data acquisition card and stored in the computer. The image reconstruction process is done off-line using the MATLAB programming language. Data from the CCD camera were processed using digital image processing to produce a single image concentration profile for the material being dropped in the pipeline.

### 3.2. Forward Model

**Figure 6.**
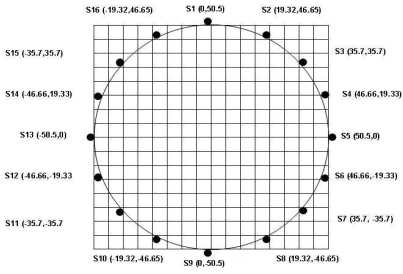
Pipe cross-section and 16 sensor locations with coordinates (x, y).

The forward model plays an important role in tomography before image reconstruction process (inverse problem). In general, a forward model deals with the theoretical aspects of the problem and is solved using mathematical modeling of the related problem. Forward modeling in electrical charge tomography imaging uses a description of the system in the sensing area. It is related to the sensitivity of sensors when a uniform three-dimensional charge, in coulomb per cubic meter (C/m^3^). Since the electrical charge tomography system gives one measurement from each of the sensors, the amount of information available is equal to the number of the sensors [15]. In this system, the cross-section of the pipe is mapped onto 16 × 16 rectangular arrays. It is equal to the number of sensors (16) and consists of 256 pixels or elements [16]. The size of the pipeline used in this system is 100 mm. Sixteen sensors are installed at equal distances around the pipe, as shown in Figure 6. This will limit the number of sensors used in the system and limit the resolution of the image. In future, it may need to increase the size of pipe to produce the better image resolution such as 32 × 32 pixels or 64 × 64 pixels.

**Figure 7.**
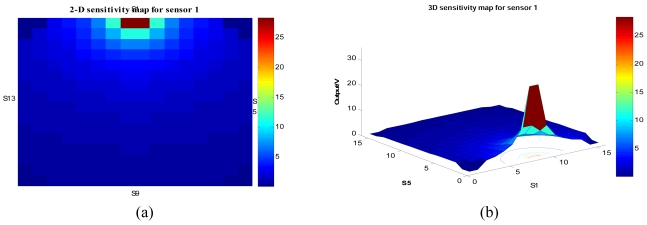
Sensitivity map for sensor 1 (a) Two dimensional (b) Three dimensional.

The sensitivity map will be generated by calculating the charge which is induce on every sensor in the system. For instance the sensitivity map of the sensor 1 with coordinates (0,50.5,0), as shown in Figure 6, will be calculated using equation (4) below:

(4) $$I_{1}=\int_{-100}^{100}dz\int_{-{\left(50.5-x^{2}\right)}^{1/2}}^{{\left(50.5-x^{2}\right)}^{1/2}}dy\int_{-50}^{50}\frac{1}{x^{2}+{\left(50.5-y\right)}^{2}+z^{2}}dx$$

where *I_1_* is the total induced charge on sensor 1. The (*x, y, z*) are the coordinates of the pixel contributing to the sensor output. The limit or border used for integration in this equation refers to the maximum and minimum values of the *x, y* and *z* axis in the measurement system. The result of the sensitivity map for sensor 1 in two and three-dimensions is shown in Figure 7.

**Figure 8.**
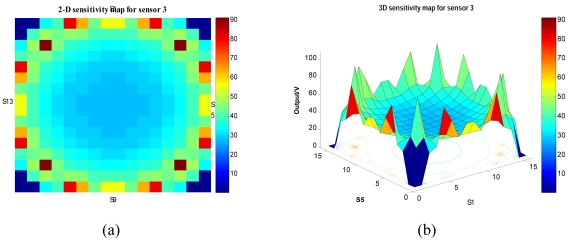
Summation sensitivity map S (a) Two-dimensional (b) three-dimensional.

The sensitivity map for the rest of the sensors is calculated using the same equation, but with some changes on the coordinates depending on the sensor coordinates, as shown in Figure 6. The summation of the complete sensitivity map, *S*, for sixteen sensors in two and three-dimensions is shown in Figure 8.

### 3.3. Inverse Problem and Image Reconstruction Algorithm in EChT

The solution of the inverse problem aims to provide an image of the charge concentration distribution within the conveyor, which would be the result of the measured sensor outputs. Image reconstruction has played an important role in electrical charge tomography systems. The data captured by the sixteen sensors is used to generate an image concentration. Three methods have been introduced before, namely the linear back-projection (LBP), filter back-projection (FBP) [16,17] and least squares [18] methods.

#### 3.3.1. Linear Back Projection (LBP)

Linear back projection is a straightforward solution which refers to the relationship between the distribution of charge, *q* and the detection of voltage by the detector as in equation (5):

(5) V=Sq

where *S* is the sensitivity map or sensitivity matrix with a dimension of 16 × 16 pixels (as discussed in Section 3.2). *V* is the measured voltage vector, and *q* is the unknown charge distribution vector that has to be solved.

To reconstruct the images in this system, the inverse problem has to be solved using equation (5). However, there are usually difficulties in calculating the inverse of matrix *S* because *S* is usually ill conditioned and its condition number is large, so even small errors in measurement may induce large errors in calculation. Thus, the concept of the general inverse matrix has to be introduced [19]. Then, equation (5) becomes *q* = *S*^-1^*V*, known as back projection. In practice, the concept of pseudo-inversion is used by assuming that *S*^-1^ = *S*^T^ [20]. In reality, S is a symmetric matrix so this would been formulated as equation (6):

(6) $$q_{\text{LBP}}=SV$$

Image concentration using LBP is obtained from the sum for each of the sixteen sensors of the product of the sensor sensitivity by its measured voltage output.

The advantages of LBP are that it is numerically simple and computationally fast because it only involves a single matrix-vector multiplication; however, the quality of reconstructed images is relatively low for complicated reconstruction objects and in some aspects it can only be considered as a qualitative algorithm [2].

#### 3.3.2. Filter Back Projection (FBP)

The major limitation of the linear back projection method arises from the non-linear sensing mechanism of the electrostatic charge transducer. However, a filter can be determined by combining it with the back projection method to provide a filtered back projection. This filter provides weighting to individual pixels, which in turn provides a uniform concentration profile when the sensor has equal outputs [12].

The filter matrix *F* is obtained by taking the maximum value of pixel (*S*_max_) in sensitivity matrix of S, divided by each value of pixel (*S*_i_), as shown in equation (7):

(7) $$\text{Filter}=\{S_{\max}/S_{i}\}$$

Filtered back projection is a result of linear back projection multiplied by filter matrix of *S*, written as equation (8):

(8) $$q_{\text{FBP}}=q_{\text{LBP}}\;\text{Filter}$$

#### 3.3.3. Least Squares Method (LS)

The least squares (LS) method provides an approximation of the solution to the inverse problem of equation when measurement error is considered. It is meant to minimize the equation (5) as shown in equation (9):

(9) $$\mathrm{Min}{\left\|Sq-V\right\|}^{2}$$

(10) $$\text{where},\;q={\left(S^{\text{T}}S\right)}^{-1}\;S^{\text{T}}V$$

The solution of equation (10) is not unique due to the fact that the matrix (*S*^T^*S*)^-1^ is not invertible. Therefore, the equation (10) is not a stable solution [21]. This is due to the ill-posed sensitivity map *S* used in this system; the difference between the first and last positive value of a singular value of matrix S is very large. Then this will contribute to the high value of the condition number of matrix S and an unstable solution [22].

#### 3.3.4. Least Square with Regularization (LSR)

Due to the problem with the singular values in the sensitivity map *S*, the method to dump small singular values in *S* has to be solved by imposing on equation (10) additional information about the solution, known as penalty term, which can optimize the problem as in equation (11):

(11) $$E(\text{q})=\arg\min{\left\|S\text{q}-V\right\|}^{2}+{ß}^{2}{\left\|R(q-q_{o})\right\|}^{2}$$

This is called the simple regularization process or Tikhonov regularization [23]. The aim of this regularization is to dampen the contribution of small singular values in the solution. The matrix *R* is a regularization matrix, which penalizes extreme changes in the parameter q removing the instability in the reconstruction [24]. The parameter *β* is called the regularization parameter. The solution of equation (11) would be written as a simple form of the standard Tikhonov (ST) where *R* = *I* (identity matrix) and assuming *q*_0_ = 0. Thus, equation (12) is introduced:

(12) $$q_{\text{ST}}={\left(S^{\text{T}}S+\beta^{2}I\right)}^{-1}S^{\text{T}}V$$

For the described algorithm, the choice of regularization is important. In general, a small value of ß gives a good approximation of the original problem but the influence of errors may make the solution physically unacceptable. Conversely, a large value of *β* suppresses the data but increases the approximation error. At present and in most cases, *β* is chosen empirically [25]. The value of the regularization parameter is obtained using a generalized cross validation (GCV) method [25,26].

The advantage of equation (12) is that it can detect two charges at separate points in the sensing area but it has a ghosting image at the adjacent points. However, FBP may be accurate in detecting the area of the charges but cannot distinguish between two separate charges in the sensing area. Therefore, the best way to solve these problems as well as the image reconstruction process, is by using the FBP image as a threshold value to erase the ghosting image in the standard Tikhonov method. The high value in standard Tikhonov method will decrease as the filter of the image reconstruction process as in equation (13). The filter matrix F_ST_ obtained by taking each value (*q*_STi_) of pixel in *q*_ST_, divided by the maximum value of pixel (*F*_STmax_) in *q*_ST_. As a result, equation (14) is used to produce the image concentration in the sensing area cin the so-called Least Squares with Regularization method (LSR):

(13) $$F_{\text{ST}}=\left\{q_{\text{STi}}/q_{\text{ST}\max}\right\}$$

(14) $$\text{Image LSR}=q_{\text{FBP}}\;F_{\text{ST}}$$

As mentioned earlier, the ill-posed problem is related to the condition number of matrix sensitivity *S* used in EChT. The singular value decomposition (SVD) will reveal all the difficulties associated with the ill conditioning of the matrix [25]. The condition number of matrix *S* is classified as ill-conditioned if the singular value of S decays gradually to zero and the ratio between the largest and the smaller nonzero singular values as high as 1 × 10^20^.

## 4. Results and Discussion

This section focuses on the functional output of the electrodynamics sensor circuit that has been designed. For the purpose of comparison, the image reconstructed by LBP, FBP and LSR methods and simulation images are presented. In additional, stability analysis using SVD was also conducted.

The data measured by the electrodynamics sensor will be used to reconstruct the image using the LBP, FBP and LSR methods. To validate the images reconstructed by these methods, a CCD camera will be used in the electrical charge tomography measurement system. The image produced by the CCD camera will be compared to the images produced by the LBP, FBP and LSR methods. The images with most similarity to the image captured by CCD camera will be identified as the best image, as well as the best method to be used to produce the image in the electrical charge tomography system.

### 4.1. Data Output from the Electrodynamics Sensor

**Figure 9.**
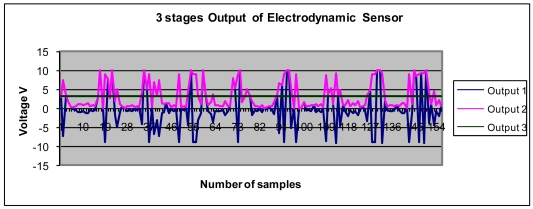
Sensor voltage outputs 1, 2 and 3.

The signal outputs from one of the electrodynamics sensors are shown in Figure 9. The measurements are performed with a sample signal at a frequency of 1 kHz. The result shows that the voltage outputs 1, 2 and 3 from the sensor are as expected. The image reconstruction process will use the output 3 of the each electrodynamics sensor, which is a DC average voltage, to construct the image concentration profile as a charge distribution in the sensing area.

### 4.2. Simulation Images

**Figure 10.**
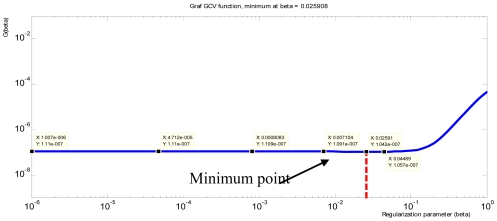
GCV curve with optimization value of *β*.

From the sensitivity matrix generated by the forward problem and predictive data obtained in the EChT measurement system, the value of the regularization parameter, *β* is calculated using the generalized cross validation (GCV) method. The GCV is a way to estimate appropriate values of the regularization parameters *β* which minimizes the GCV function as in equation (15) [25]:

(15) $$G=\frac{{\left\|Sq_{ST}-V\right\|}_{2}^{2}}{{\left(\text{trace}\left(I_{m}-SS^{I}\right)\right)}^{2}}$$

where S*^I^* is a matrix which produces the regularized solution *q*_ST_ when multiplied with *V, i.e., S^I^* = (*S*^T^*S* + (*β*^2^)*I*)*S*^T^. GCV method indeed seeks to balance the perturbation and regularization errors and thus, in turn, is related to the corner of L-curve [4]. Figure 10 shows the GCV curve with optimization value for regularization parameter obtained using Matlab tools programming [25]. From the graph, the minimum point is 0.025908 as labeled in Figure 10.

**Figure 11.**
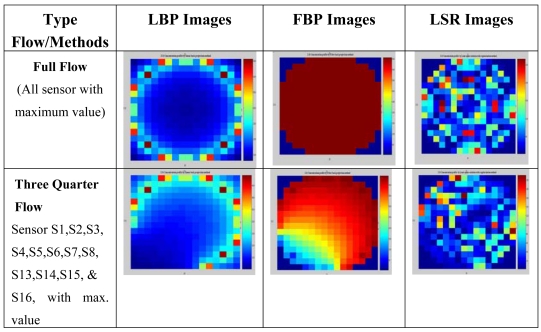

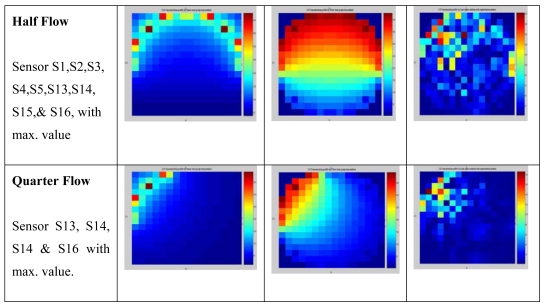
Simulation images for different types of flow pattern using LBP, FBP and LSR methods.

This value is used to generate the image concentration for the LSR method. Figure 11 shows the image reconstructed for different types of flow pattern such as full flow, three quarter flow, half flow and quarter flow. These patterns are reconstructed using predicted values of electrodynamic sensor output by the LBP, FBP and LSR methods respectively. In general, Figure 11 shows that image reconstructed by the LBP method with high concentration areas is focused on the sensors itself (refer to Figure 6). The FBP method shows that the high-concentration area is focused around particular sensors with the huge point of charges and the concentration value is reduced towards the centre of the pipe. The LSR method produces the high concentration area as detected by the LBP and FBP methods. Its concentration is homogenously scattered around the pipe with different concentration values and many charges are present in the sensing area. However, the LBP method is not suitable for use in this system because of the nonlinearity of the sensor mechanism. On the other hand, the FBP method may be accurate in detecting the high concentration area but it cannot differentiate between two or many charges present in the sensing area. In conclusion, the image reconstructed by the LSR method is accurate in detecting high concentration areas and has the capability to differentiate charges in the sensing area. Table 1 shows the condition number of different types of flow pattern. This condition number is obtained from numerical analysis using the SVD method. It shows that the LSR method produces the smallest value of the condition number as compared to other methods. It means that image reconstructed by LSR method is more stable than the other methods.

### 4.3. Experimental Images

This section will discuss the result processed from the data being measured in electrical charge tomography measurement system using an electrodynamic sensor and CCD camera. For comparison, two types of flow rate had been chosen *i.e.*, 10 and 50 flow rate speed indications, as recorded by the system.

#### 4.3.1. Flow Rate with 10-Indication Speed

**Figure 12.**
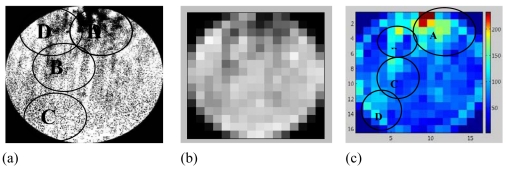
Image recorded by CCD Camera (a) Real Image Captured (b) Real image resized to 16 × 16 pixels with grey color (c) Real image resized to 16 × 16 pixels with color mode.

From the experiments conducted using the apparatus shown in Figure 5, several images had been captured to verify images from LBP, FBP and LSR image reconstruction algorithm. Figure 12 shows the image captured by the CCD Camera with three different modes. Figure 12(a) shows the original image after identification of the sensing area for material flow in the pipeline. Figure 12(b) is the image of Figure 12(a) after being resized to a 16 by 16 matrix with gray color and Figure 12(c) is the image of Figure 12(b) after being converted to color mode-using MATLAB.

**Figure 13.**
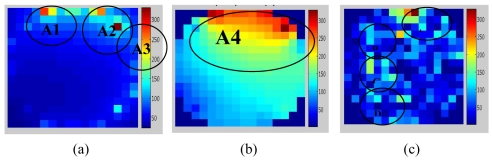
Image reconstructed by (a) LBP (b) FBP and (c) LSR methods.

The Figure 13 shows image reconstructed by the LBP, FBP and LSR methods based on the data captured by the electrodynamics sensor at the same time the CCD camera recorded the video (as shown in Figure 12).

In Figure 13(a), the image reconstructed using the LBP method shows that the high concentration values of the image are not focusing on the area shown by the image captured by the CCD camera [as shown in Figure 12(c)]. Besides, it focuses around the sensor located near the real image labeled as A1, A2 and A3. It means that LBP method is not suitable for reconstructing the image for electrical charge tomography because of its non-linearity to the sensing mechanism.

Figure 13(b) shows that the image reconstructed using the FBP method is accurate in detecting the high concentration area labeled as A4. However, there is a larger point image compared to the one in Figure 12(c). Another problem with FBP is that a high concentration exists near the sensor which reduces the value away toward the centre. As a result, two or many charges in the sensing area cannot be differentiated by FBP.

In Figure 13(c), the high image concentration area for the LSR method is similar to the real image recorded by the CCD camera area, labeled as A, B, C and D in Figure 12(c). It means that LSR method is capable of differentiating two or many charges in the sensing area, as shown in Figure 13(c). It shows that charge concentrations areas detected by the LSR method are scattered around the sensing area as well as around the image presented by the CCD Camera. Although the number of pixels area for high concentration is different, the pattern is the same. This is due to the problem in selecting the sensing area in the pipeline cross-section during image processing. This can be avoided by using a marking system around the pipe with sensor location as a reference point.

#### 4.3.2. Flow Rate with 50-Indication Speed

**Figure 14.**
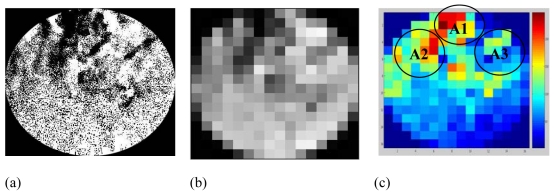
Image recorded by CCD Camera (a) Real Image Captured (b) Real image resized to 16 × 16 pixels with grey color (c) Real image resized to 16 × 16 pixels with color mode.

Figure 14 shows the images captured by the CCD camera with different processing modes as discussed in 4.3.1. Figure 14(a) is the real image with identified sensing area. Figure 14(b) is the image of Figure 14(a) after being resized to a 16 by 16 matrix with gray color. Figure 14(c) is the image of Figure 14(b) after converted to color mode-using MATLAB.

**Figure 15.**
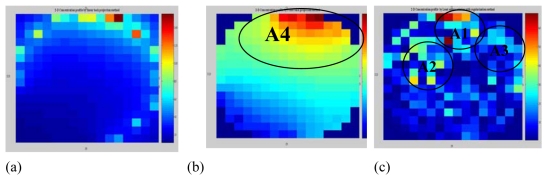
Image reconstruction by (a) LBP (b) FBP and (c) LSR methods.

Figure 15 shows the image reconstructed by the LBP, FBP and LSR methods based on data captured using the electrodynamics sensor at the same time the CCD camera recorded the video (as shown in Figure 14).

Figures 14 and 15 shows an almost identical result is to the one obtained in 4.3.1. The LSR method produces a similar image to the image produces by the CCD camera. It shows that the charge concentrations area detected by the LSR method are scattered around the sensing area as well as image presented by the CCD Camera as shown in Figure 14(c). Thus, images in Figure 15(a) and (b) obtained by the LBP and FBP methods are rejected. Table 2 shows the condition number for two types of flows as discussed in 4.3.1 and 4.3.2.

It shown that LSR method produced the smallest value of the condition number compared to the LBP and FBP methods. It means that image reconstructed by the LSR method is more stable.

## 5. Conclusions

Electrical charge tomography (EChT) is considered as one of the promising tomography process technologies due to its advantages such as high speed, low cost and non-intrusive sensors, especially for dilute flow condition where the solid-air ratio is low. As a result, the measured concentration data using an electrodynamics sensor enjoys a large degree of immunity from the effects of solid acceleration. The success of application in image reconstruction depends greatly on the precision of the image reconstruction algorithm. In this paper, an image reconstruction algorithm – least squares with regularization (LSR) has solved some problems in EChT image reconstruction such as ill-condition of matrix S and accuracy of the reconstructed image. With an imposed regularization method and empirically chosen regularization parameters, results show that the proposed algorithm is efficient in overcoming the problems of stability and accuracy of the image being reconstructed. In addition, more work such as computation of image errors and data measurement errors should be done in validating the proposed algorithm. The ill-posed sensitivity matrix should also be improved. In terms of electrodynamics sensor improvement, the sensor of spatial resolution such as diameter, length and number of sensors to be used should also be more rigorously considered. This will directly affect the data measurement and the quality of the reconstructed images. To enable EChT technology to be used in real industry environment, more work on hardware and software systems should be carried out and the image reconstruction algorithm should be further developed.

## Figures and Tables

**Table 1.** Images condition number for different types of flow.

**Flow rate/Condition number**	**LBP (condition number)**	**FBP (condition number)**	**LSR (condition number)**

**Full Flow**	8.06 × 10^17^	2.908 × 10^17^	125.60
**Three quarter Flow**	1.79 × 10^6^	3.13 × 10^6^	187.07
**Half Flow**	3.14 × 10^18^	3.35 × 10^17^	302.34
**Quarter flow**	2.13 × 10^6^	3.65 × 10^5^	432.48

**Table 2.** Images condition number for two types of flow.

**Flow rate/Condition number**	**LBP (condition number)**	**FBP (condition number)**	**LSR (condition number)**

**10**	7.62 × 10^5^	5.15 × 10^5^	219.99
**50**	7.96 × 10^6^	2.00 × 10^6^	283.73
